# Chemogenetic Suppression of the Subthalamic Nucleus Induces Attentional Deficits and Impulsive Action in a Five-Choice Serial Reaction Time Task in Mice

**DOI:** 10.3389/fnsys.2020.00038

**Published:** 2020-06-30

**Authors:** Tadaaki Nishioka, Kosuke Hamaguchi, Satoshi Yawata, Takatoshi Hikida, Dai Watanabe

**Affiliations:** ^1^Department of Biological Sciences, Graduate School of Medicine, Kyoto University, Kyoto, Japan; ^2^Laboratory for Advanced Brain Functions, Institute for Protein Research, Osaka University, Suita, Japan

**Keywords:** subthalamic nucleus, attention, impulsivity, 5-choice serial reaction time task, DREADD

## Abstract

The subthalamic nucleus (STN), a key component of the basal ganglia circuitry, receives inputs from broad cerebral cortical areas and relays cortical activity to subcortical structures. Recent human and animal studies have suggested that executive function, which is assumed to consist of a set of different cognitive processes for controlling behavior, depends on precise information processing between the cerebral cortex and subcortical structures, leading to the idea that the STN contains neurons that transmit the information required for cognitive processing through their activity, and is involved in such cognitive control directly and dynamically. On the other hand, the STN activity also affects intracellular signal transduction and gene expression profiles influencing plasticity in other basal ganglia components. The STN may also indirectly contribute to information processing for cognitive control in other brain areas by regulating slower signaling mechanisms. However, the precise correspondence and causal relationship between the STN activity and cognitive processes are not fully understood. To address how the STN activity is involved in cognitive processes for controlling behavior, we applied Designer Receptors Exclusively Activated by Designer Drugs (DREADD)-based chemogenetic manipulation of neural activity to behavioral analysis using a touchscreen operant platform. We subjected mice selectively expressing DREADD receptors in the STN neurons to a five-choice serial reaction time task, which has been developed to quantitatively measure executive function. Chemogenetic suppression of the STN activity reversibly impaired attention, especially required under highly demanding conditions, and increased impulsivity but not compulsivity. These findings, taken together with the results of previous lesion studies, suggest that the STN activity, directly and indirectly, participates in cognitive processing for controlling behavior, and dynamically regulates specific types of subprocesses in cognitive control probably through fast synaptic transmission.

## Introduction

Executive function—cognitive control of behavior—depends on the integrative properties of interconnected circuits consisting of the cerebral cortex and subcortical structures. Among the cortico-subcortical circuitry, the subthalamic nucleus (STN), which is mainly composed of glutamatergic neurons (Barroso-Chinea et al., 2007; Koshimizu et al., 2013) and relays cortical activity to subcortical structures as a component of the basal ganglia (Parent and Hazrati, 1995; Nambu et al., 2002), is postulated to play a pivotal role in cognitive processes for controlling behavior (Baunez and Robbins, 1997; Baunez et al., 2001; Aron and Poldrack, 2006; Zaghloul et al., 2012; Weintraub and Zaghloul, 2013). Despite increasing interest in the physiological function and clinical relevance of the STN, however, the precise correspondence and causal relationship between the STN activity and cognitive processes are not fully understood.

The STN, directly and indirectly, receives information from various cortical areas, including the prefrontal cortex (PFC), through two major distinct types of afferent synaptic inputs: excitatory glutamatergic from the cortical neurons and inhibitory GABAergic from the pallidal neurons (Parent and Hazrati, 1995; Kolomiets et al., 2001; Haynes and Haber, 2013; Kita et al., 2014), which are organized as the hyperdirect (Nambu et al., 2002) and indirect pathway (Alexander and Crutcher, 1990) in the cortico-basal ganglia circuit, respectively. In turn, the STN projects to brain areas involved in both motor and cognitive functions, such as the globus pallidus (GP), entopeduncular nucleus (EPN), substantia nigra pars reticulata (SNr), and substantia nigra pars compacta (SNc) in the basal ganglia and other subcortical structures (Kanazawa et al., 1976; Parent and Hazrati, 1995; Iribe et al., 1999; Koshimizu et al., 2013). Furthermore, since the STN has been identified as a potential target of deep brain stimulation (DBS) therapy to treat symptoms of Parkinson’s disease (PD; Limousin et al., 1995; Benabid et al., 2009), numerous studies in PD patients and animal models have demonstrated that DBS manipulation of the STN not only improves motor impairments but also alters non-motor cognitive functions (Desbonnet et al., 2004; van den Wildenberg et al., 2006; Baunez et al., 2007; Ballanger et al., 2009; Jahanshahi et al., 2015). Thus, anatomical connectivity of the STN and functional manipulation of its activity support the idea that the STN functions as an integrative node that links cognitive processing with motor and other functions, and the activity state of the STN neurons is critical for cognitive control (Aron and Poldrack, 2006; Zaghloul et al., 2012; Weintraub and Zaghloul, 2013).

STN lesion studies have so far demonstrated that the ablation of the STN neurons impairs various cognitive processes for controlling behavior (Baunez and Robbins, 1997; Baunez et al., 2001). On the other hand, permanent STN inactivation decreases the expression of GAD67 in the GP (Delfs et al., 1995) and cytoplasmic membrane dopamine transporter in the striatum (Str; Schweizer et al., 2014), and facilitates phosphorylation of Akt and ribosomal protein S6 (rpS6) in the SNc (Luke Fischer et al., 2017), suggesting that the STN activity affects signal transduction and gene expression profiles influencing plasticity of the GP, Str, and SNc. Because these basal ganglia components are postulated to play essential roles in cognitive processing, the STN may not only directly regulate cognitive information through fast synaptic transmission but also indirectly affect cognitive processing in other brain areas through slower signaling mechanisms.

To further dissect the fast action of the STN on cognitive functions, it is important to manipulate the activity of the STN neurons immediately and reversibly. Conventional DBS manipulation can be temporally controlled. However, the effect of such DBS targeting the STN is intrinsically complicated. High-frequency electrical stimulation can sometimes facilitate the activity of the STN neurons, but other times inhibit their excitability, depending on stimulation parameters including intensity, frequency, distance and orientation of the electrical stimuli (McIntyre et al., 2004; Welter et al., 2004; Ledonne et al., 2012; Shehab et al., 2014; Ramasubbu et al., 2018). DBS also affects the activity of both the excitatory and inhibitory afferents to the STN from the cerebral cortex and GP, respectively (McIntyre et al., 2004; Gradinaru et al., 2009; Li et al., 2012). Furthermore, the effect of electrical stimulation by electrodes implanted in the STN can spread beyond, into neighboring brain areas such as the zona incerta (ZI), which is also profoundly involved in cognitive functions (Mitrofanis, 2005; Zhang and van den Pol, 2017).

The Designer Receptors Exclusively Activated by Designer Drugs (DREADD)-based chemogenetic approach (Armbruster et al., 2007; Wulff and Arenkiel, 2012) can provide an effective solution to circumvent issues arising from uneven effects of electrical stimulation on activity of the individual STN neurons and its off-target effects. The engineered G protein-coupled receptor hM4Di is a mutated human M_4_ muscarinic acetylcholine receptor with amino acid substitutions that abolish receptor affinity for the physiological ligand acetylcholine but allow receptor binding and subsequent activation by a pharmacologically inert compound clozapine-N-oxide (CNO; Armbruster et al., 2007). Thus, unlike electrical stimulation, CNO-binding to hM4Di can robustly activate the Gi signaling pathway leading to selective and reversible inhibitory action on the hM4Di-expressing neurons (Ray et al., 2011; Wulff and Arenkiel, 2012).

In this study, we selectively introduced hM4Di-DREADD in STN neurons by a combinatorial gene expression system utilizing an adeno-associated virus (AAV) vector with double-floxed inverted open reading frames (DIO), which is transcriptionally activated by Cre-mediated recombination (Schnütgen et al., 2003), and a paired-like homeodomain transcription factor 2-Cre (Pitx2-Cre) mouse line as a Cre-driver (Liu et al., 2003; Martin et al., 2004; Skidmore et al., 2008; Schweizer et al., 2014, 2016). To address how suppression of the STN activity affects cognitive processing, we subjected mice selectively expressing hM4Di in the STN neurons to a five-choice serial reaction time task (5-CSRTT), which has been developed to quantitatively assess executive function in rodent (Bari et al., 2008; Mar et al., 2013) and analyzed their behavioral performance in the presence or absence of CNO (Carli et al., 1983; Liu et al., 2003; Lein et al., 2007; Koike et al., 2015).

## Materials and Methods

### Animals

Heterozygous 129S-Pitx2-Cre^tm4(Cre)Jfm^/Mmucd mice (RRID:MMRRC_000126-UCD; MMRRC; Liu et al., 2003), maintained in a C57BL/6N background, were used in all experiments (8–10-weeks old male). Animals were housed on a 12-h light/dark cycle. Behavioral studies were conducted during the dark cycle. Mice were kept on water restriction during behavioral testing. All experiments conformed to the guidelines of the National Institutes of Health experimental procedures and were approved by the Animal Care and Use Committee of Kyoto University.

### Viral Injection

Mice were anesthetized with ketamine (100 mg/kg) and xylazine (20 mg/kg). 400 nl of rAAV5-hSyn-DIO-hM4Di(Gi)-mCherry (5.1 × 10^12^ GC/ml, UNC) or rAAV5-hSyn-DIO-mCherry (5.2 × 10^12^ GC/ml, UNC) were stereotaxically injected using a Nanoject III instrument (Drummond) at a rate of 100 nl/min (coordinates in mm: AP −1.90, ML ± 1.70 from bregma, and DV −4.60 and −4.25 from brain surface). The injection pipette remained in place for 5–10 min to reduce backflow.

### Five-Choice Serial Reaction Time Task (5-CSRTT)

#### Apparatus

Training and testing were conducted in a Bussey-Saksida touchscreen chamber (Lafayette Instrument). A black plastic mask with five windows (40 × 40 mm^2^ spaced, 9 mm apart, 16 mm above the floor) was placed in front of the touchscreen. ABET II and WhiskerServer software (Lafayette) were used to control the operant system and data collection.

#### Pretraining

As the first phase (3 days), mice were habituated to the chamber in 40-min sessions. Diluted condensed milk (7 μl, Morinaga Milk) was dispensed in the food magazine every 10 s. In the following phase (1 day), a stimulus was randomly displayed in one of the 5 windows. After a 30-s stimulus presentation, the milk reward (20 μl) was delivered with a tone (3 kHz) and magazine light. When mice collected the reward, the magazine light went out, and the next trial commenced (30 trials, or up to 60 min) with a new stimulus after a 20-s intertrial interval (ITI). In the next phase, stimuli were randomly displayed in one of 5 windows, and mice were obligated to touch the stimulus to receive a reward. In the final phase of the pretraining, when a blank window was touched, mice were punished with a 5-s time-out. After reaching criterion (77% correct for two consecutive days), mice moved on to basic training.

#### Basic Training

The 5-CSRTT was similar to one that was described (Carli et al., 1983; Koike et al., 2015). Mice were tested 5–6 days per week (60 trials per day, or up to 60 min). Each trial was initiated after mice nose-poked in the magazine. The stimulus was delivered after a 5-s delay period. If a mouse touched a window during the delay period, the response was recorded as a premature response, and the mouse was punished with a 5-s time-out (house light on). The stimulus duration (SD) was set to 4 s, followed by a limited holding period of 5 s. Responses during the stimulus presence and limited holding period were recorded as correct responses and rewarded with a tone. A response to any other window, or failure to respond during the stimulus presence or the limited holding period, was recorded as an incorrect response or omission, respectively, and punished with a 5-s time-out. Additional responses to the touchscreen after a correct response before collecting the reward were recorded as perseverative responses. Once the performance stabilized at 4-s SD (>80% accuracy, <20% omissions for three consecutive days), SD was reduced to 2 s. After reaching criterion at 2-s SD (>80% accuracy, <20% omissions for two consecutive days), animals were trained for an additional 2 days with intraperitoneal vehicle injection.

#### Probe Test

Animals were tested with increased attentional demand by reducing SD to 2, 1.5, 1, and 0.8 s. Vehicle (10 ml/kg of 0.5% DMSO in saline) at day 1 and 3 or CNO (10 mg/kg diluted with the vehicle, Sigma Aldrich) at day 2 and 4 was intraperitoneally administered 30 min before the session. Response accuracy (correct trials divided by correct plus incorrect trials, recorded as a percent), omissions (omitted trials divided by total trials, in %), premature responses, perseverative responses (per choice), and latencies to correct response, incorrect response, premature response, and reward collection after each correct response were monitored.

### Electrophysiology

Mice were deeply anesthetized and transcardially perfused with ice-cold *N*-methyl-D-glucamine (NMDG)-based artificial cerebrospinal fluid (ACSF) containing 93 mM NMDG, 2.5 mM KCl, 1.2 mM NaH_2_PO_4_, 30 mM NaHCO_3_, 10 mM MgSO_4_, 0.5 mM CaCl_2_, 20 mM HEPES, 5 mM Na-ascorbate, 3 mM Na-pyruvate, 2 mM thiourea, and 25 mM D-glucose equilibrated with 95% O_2_ and 5% CO_2_. Sagittal forebrain slices (250 μm) were cut in ice-cold NMDG-ACSF using a Leica VT1200S microtome. The slices were incubated initially in NMDG-ACSF for 15 min at 34°C, followed by a 60 min incubation at 21°C in standard ACSF (125 mM NaCl, 2.5 mM KCl, 1.25 mM NaH_2_PO_4_, 26 mM NaHCO_3_, 1 mM MgCl_2_, 2 mM CaCl_2_, 20 mM D-glucose equilibrated with 95% O_2_ and 5% CO_2_). Reporter-expressing cells were identified using an Olympus microscope equipped with epifluorescence illumination and infrared differential interference contrast (IR-DIC) optics. Whole-cell patch-clamp recordings were obtained using borosilicate glass pipettes (4–7 MΩ) filled with an internal solution (120 mM K-gluconate, 2 mM NaCl, 5 mM MgCl_2_, 10 mM HEPES, 2 mM EGTA, 2 mM ATP-Na_2_, 0.3 mM GTP-Na_3_ adjusted to pH 7.30). Immediately after the 5-min break-in process, the membrane potential was held at approximately −60 mV by holding the current injection, and the minimum amplitude of a step current that elicited an action potential (AP) was determined. Another 5 min after the establishment of the whole-cell configuration, CNO (10 μM) was bath-applied. The data obtained 1–2 min before CNO application and those obtained 3–4 min after CNO application were used for analysis. All the recordings were performed in the presence of AMPA-, NMDA-, and GABA_A_-receptor antagonists (10 μM NBQX, 50 μM D-APV, and 20 μM bicuculline, respectively; Tocris). The liquid junction potential (11.8 mV) was corrected for analysis.

### Tissue Preparation, Immunohistochemistry, and Image Analysis

Animals were deeply anesthetized and transcardially perfused with 0.01 M PBS followed by 4% paraformaldehyde (PFA) in 0.1 M PB (pH 7.4). Brains were removed and post-fixed with 4% PFA at 4°C for 2 days. After cryoprotection, brains were embedded in OCT compound and cryosectioned (thickness: 40 μm). Sections were permeabilized and blocked with 0.1% Triton X-100 and 10% normal goat serum (NGS) in PBS for 60 min at room temperature, then incubated with 1:500-diluted primary antibodies in PBS containing 10% NGS overnight at 4°C. The primary antibodies used for detecting neuronal cells and enhancing mCherry fluorescence were mouse anti-NeuN (Millipore) and rabbit anti-DsRed (Clontech) antibodies, respectively. After three washes, the sections were incubated for 1 h at room temperature with 1:500-diluted secondary antibodies in PBS containing 0.5% NGS. The secondary antibodies used were Alexa Fluor 488-conjugated goat anti-mouse IgG and Alexa Fluor 555-conjugated goat anti-rabbit IgG antibodies (Life Technologies). After three washes, sections were incubated with DAPI (0.2 μg/ml) and mounted. Stitched images were acquired using a Keyence BZ-X710 microscope. For cell counting, images of the anterior, middle, and posterior STN were obtained from three coronal sections (section level −1.82 mm, −2.06 mm, and −2.30 mm from bregma, respectively; *n* = 6 mice) using a confocal laser microscope (Olympus, FV1200), and the number of Neu-N positive cells and mCherry-expressing Neu-N positive cells were analyzed using the built-in cell counter plugin of NIH ImageJ software.

### Statistical Analyses

Prism (Graphpad) software was used for statistical analyses. The electrophysiological data were analyzed using a two-way repeated-measures analysis of variance (RM ANOVA) with Group (hM4Di, mCherry) and Drug Treatment (before, after CNO) as within-subjects factors, followed by *post hoc* Bonferroni’s multiple comparisons test when F-ratios of the interaction were significant (*p* < 0.05). The normality test (Anderson-Darling test or Shapiro-Wilk test) was applied to assess the normality of the distribution for the 5-CSRTT data (*p* < 0.05). Considering that accuracy (%), omission (%), perseverative responses, and latencies to correct response, incorrect response and premature response were normally distributed, and premature responses were lognormally distributed, the 5-CSRTT data were analyzed using two-way RM ANOVA with Group (hM4Di, mCherry) and Drug Treatment (vehicle, CNO). To evaluate the effect of SD in 5-CSRTT, the data were analyzed using two-way ANOVA with Group (hM4Di, mCherry) and SD (0.8, 1.0, 1.5, 2.0 s). Frequency distributions were compared using the Kolmogorov-Smirnov test. All data are expressed as means ± SEM.

## Results

### Selective and Efficient Genetic Manipulation of the STN Neurons

**Figure 1:**
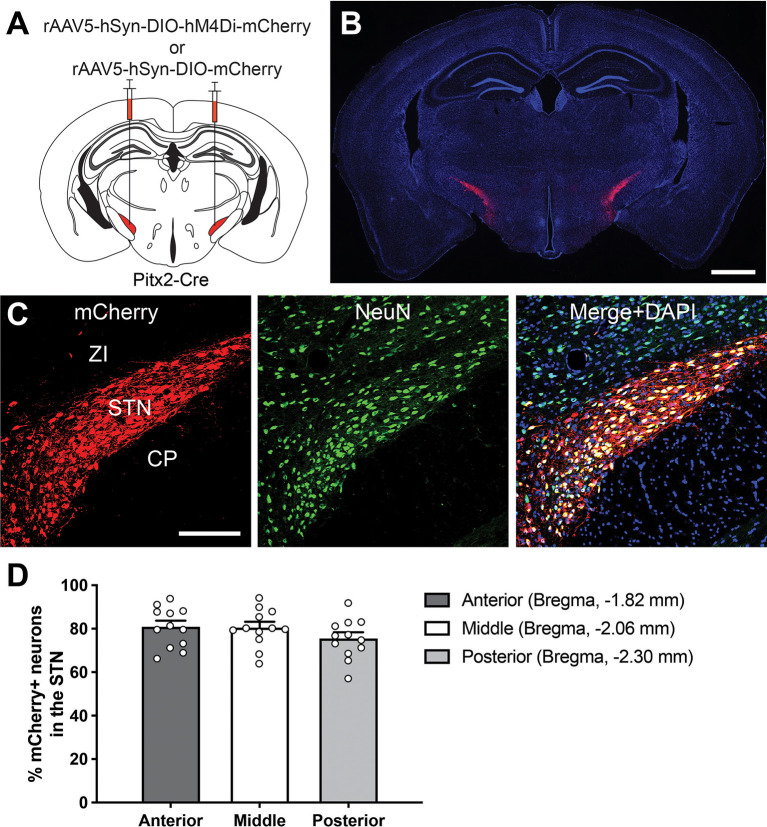
Selective and efficient genetic manipulation of STN neurons. **(A)** A schematic illustrating adeno-associated virus (AAV) vector injection into the Pitx2-Cre mouse brain. The drawing is adapted from The Mouse Brain in Stereotaxic Coordinates (Paxinos and Franklin, 2004). **(B)** A representative coronal brain section after rAAV5-hSyn-DIO-mCherry injection. The reporter fluorescence of mCherry enhanced by immunolabeling (red) was observed in the STN. DAPI (blue) was used for the counterstain. Scale bar, 1 mm. **(C)** Fluorescence imaging of mCherry enhanced by immunolabeling (red) and NeuN immunoreactivity (green). DAPI (blue) was used for the counterstain. Scale bar, 200 μm. **(D)** Quantification of the percentage of total mCherry-labeled neurons in the anterior, middle, and posterior STN (12 areas each from six mice). CP, cerebral peduncle; STN, subthalamic nucleus; ZI, zona incerta.

To genetically manipulate the STN neurons without affecting neighboring brain areas, we applied a combinatorial gene expression system utilizing an AAV-DIO vector, which is transcriptionally activated by Cre-mediated recombination (Figure 1A), and the Pitx2-Cre mouse line as a Cre-driver (Liu et al., 2003; Martin et al., 2004; Skidmore et al., 2008; Schweizer et al., 2014, 2016). To characterize Cre-mediated gene expression in our system, we stereotaxically injected the rAAV5-hSyn-DIO-mCherry into the bilateral STN of Pitx2-Cre mice (Figure 1A). Two weeks after AAV injection, cells with mCherry signals enhanced by immunofluorescence were densely distributed within the STN (Figure 1B), and mCherry-expressing axons were strongly observed in the STN target structures, the GP, SNr, and EPN (Supplementary Figure S1). Although neuronal marker NeuN-immunoreactive cells were also distributed in the ZI, mCherry signals were restricted to the NeuN-immunoreactive population in the STN (Figure 1C). These observations indicate that the reporter protein was selectively expressed in the STN neurons. Expression efficiency of the reporter in the anterior, middle, and posterior STN were 81.1 ± 2.6%, 80.7 ± 2.5% and 75.7 ± 2.7%, respectively (2,730 out of 3,449 cells, *n* = 12 in six mice; Figure 1D). Thus, the combinatorial expression system using the AAV-DIO vector and Pitx2-Cre mice enables a highly selective and efficient genetic manipulation of the STN neurons.

### CNO-Induced Activation of hM4Di Suppresses the Activity of the STN Neurons

**Figure 2:**
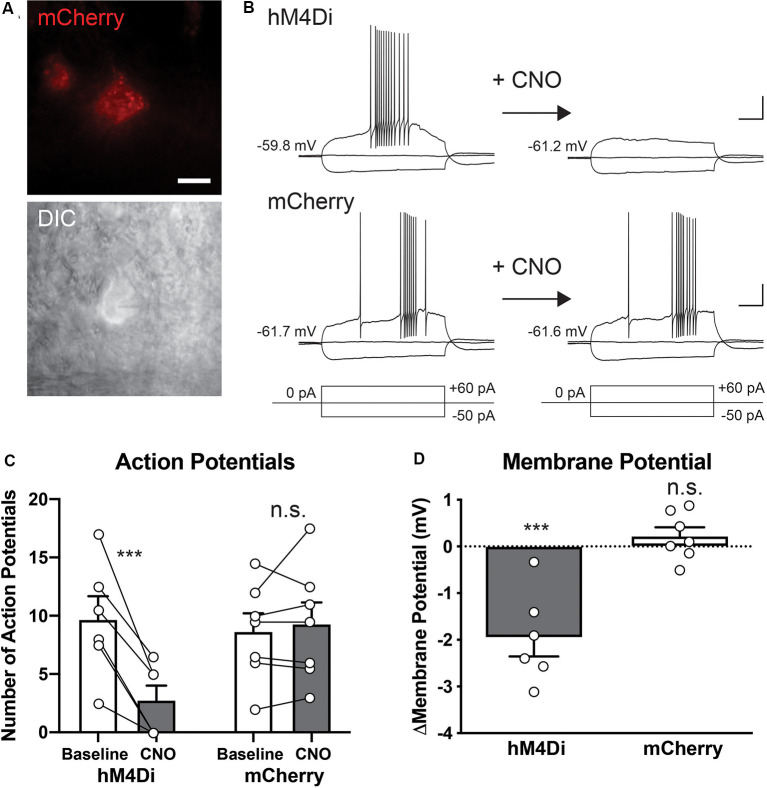
Clozapine-N-oxide (CNO)-induced activation of hM4Di suppresses the activity of STN neurons. **(A)** hM4Di-expressing STN neurons were identified by mCherry fluorescence (red), and whole-cell recordings were performed under IR-DIC optics. Scale bar, 10 μm. **(B)** Representative voltage responses of hM4Di- and mCherry-expressing STN neurons to current injection steps (+60 and −50 pA for 500 ms) before and after CNO application. Scale bars, 20 mV, 100 ms. **(C)** CNO application decreased the number of action potentials (AP) in hM4Di-expressing STN neurons but did not influence mCherry-expressing neurons. **(D)** CNO application shifted the membrane potentials to the hyperpolarized direction in hM4Di-expressing STN neurons but did not affect mCherry-expressing neurons. ***p < 0.001, n.s., not significant. Data are mean ± SEM.

To validate the effect of hM4Di activation on the intrinsic excitability of the STN at a single-cell level, we performed patch-clamp experiments. Two weeks after injection of rAAV5-hSyn-DIO-hM4Di-mCherry or rAAV5-hSyn-DIO-mCherry into the STN of the Pitx2-Cre mice, acute brain slices were prepared. hM4Di- or mCherry-expressing cells in the slice preparation were identified by epifluorescence illumination, and whole-cell recording in current-clamp mode was conducted (Figure 2A). The recorded cells were held nearly at −60 mV by holding the current injection, and the membrane potentials with hyperpolarizing, holding, and depolarizing current injection before and after CNO application were analyzed (Figure 2B). The effect of CNO on the number of evoked APs was selective to hM4Di-expressing cells (Group × Treatment interaction, *F*_(1,11)_ = 24.30, *p* = 0.0004). CNO application significantly decreased the number of APs generated by depolarizing current injection (+60 pA for 500 ms) in hM4Di-expressing cells (Figure 2C, 9.7 ± 2.0 APs before vs. 2.8 ± 1.3 APs after CNO application, *n* = 6 cells, *p* = 0.0001) but not mCherry-expressing cells (8.6 ± 1.6 APs before vs. 9.3 ± 1.9 APs after CNO application, *n* = 7 cells, *p* > 0.99). After CNO application, the basal membrane potentials shifted to the hyperpolarized direction in hM4Di-expressing cells (Figure 2D, delta V_m_ = −1.95 ± 0.40 mV, *n* = 6 cells, Group × Treatment interaction, *F*_(1,11)_ = 26.41, *p* = 0.0003; before vs. after CNO, *p* = 0.0001), but not in mCherry-expressing cells (delta V_m_ = +0.22 ± 0.19 mV, *n* = 7 cells, before vs. after CNO, *p* = 0.92). This hyperpolarization effect of CNO was also consistent with the increase of minimal current amplitude, which is required for the generation of APs, after CNO application observed in hM4Di-expressing cells (Supplementary Figure S2, +7.50 ± 0.27 pA, *n* = 6, *p* = 0.012), but not in mCherry-expressing cells (−1.43 ± 1.43 pA, *n* = 7 cells, *p* > 0.99). Together, these results indicate that CNO treatment selectively decreases the excitability of the hM4Di-expressing STN neurons.

### A Quantitative Behavioral Paradigm for Assessing Cognitive Control

**Figure 3:**
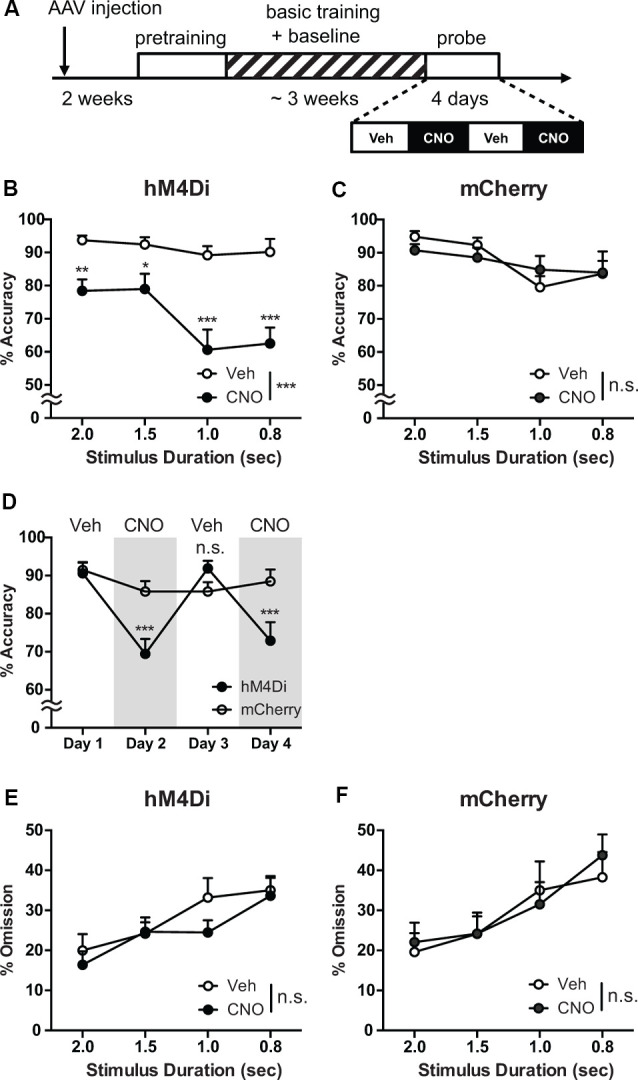
Chemogenetic reversible suppression of the STN neuronal activity impairs attentional performance. **(A)** Experimental design. **(B)** In hM4Di mice, CNO administration significantly decreased response accuracy across all stimulus duration (SD), and the impairment produced by CNO treatment was more severe with shortened SD (*n* = 8 mice). **(C)** In mCherry mice, no significant effect of CNO treatment on response accuracy was observed (*n* = 8 mice). **(D)** The decrease in response accuracy was selectively observed in hM4Di mice with CNO treatment at day 2 and 4. **(E,F)** No significant effect of CNO application on omission was detected in both hM4Di and mCherry mice (*n* = 8 mice per group). ***p < 0.001, **p < 0.01, *p < 0.05, n.s., not significant. Data are mean ± SEM.

To address how the suppression of the STN activity affects cognitive control, we analyzed the behavioral performance in a 5-CSRTT with and without CNO administration. Two weeks after injection of rAAV5-hSyn-DIO-hM4Di-mCherry or rAAV5-hSyn-DIO-mCherry into the STN of Pitx2-Cre mice (hM4Di or mCherry mice, *n* = 8 respectively), we started the pretraining, followed by the basic training, in which mice are required to withhold responses during the delay period (Figure 3A). In the 5-CSRTT, impulsivity is assessed by monitoring the number of premature responses and latency to premature responses. In the course of training, as indicated by the changes in both indices (Supplementary Figure S3), mice adaptively acquired response control. No significant difference in task performance between the hM4Di and mCherry mice was observed (Supplementary Figure S4), indicating that hM4Di does not affect the STN function in the absence of CNO.

### Chemogenetic Reversible Suppression of the STN Neuronal Activity Impairs Attentional Performance

After completing the basic training, mice were challenged in a probe test with increased attentional demand by reducing the SD to 2, 1.5, 1, and 0.8 s in a pseudorandomized order. During the probe test, CNO (10 mg/kg) or vehicle solution was administered before each session according to the schedule as shown in Figure 3A.

In the 5-CSRTT, impairment of attention processing is quantitatively measured as a decrease in response accuracy and an increase in omission (Bari et al., 2008; Mar et al., 2013). In hM4Di mice, compared with vehicle treatment, CNO administration significantly decreased response accuracy across all SDs (Figure 3B, Treatment effects, *F*_(1,7)_ = 40.5, *p* = 0.0004). In contrast, no significant effect of CNO treatment on response accuracy was observed in mCherry mice (Figure 3C, *F*_(1,7)_ = 0.048, *p* = 0.83). Decreasing SD deteriorates the response accuracy in both hM4Di and mCherry mice (hM4Di, *F*_(3,21)_ = 8.96, *p* = 0.0005; mCherry, *F*_(3,21)_ = 4.15, *p* = 0.019). Such deterioration tendency of response accuracy observed in shorter SDs was more severe in CNO-treated hM4Di mice (Treatment × SD interaction, *F*_(3,21)_ = 3.22, *p* = 0.043) but not detectable in mCherry mice (*F*_(3,21)_ = 1.31, *p* = 0.30).

To confirm the reversibility of DREADD, we alternated CNO and vehicle administration over 4 days (Figure 3D). The decrease in response accuracy was selectively observed on CNO treatment days (day 2 and day 4) in hM4Di mice (Group × Day interaction, *F*_(3,42)_ = 9.43, *p* < 0.0001; day 2 and day 4 vs. day 1, *p* < 0.0001), whereas we could detect no difference between vehicle days (day 1 and day 3) in hM4Di (*p* > 0.99). Furthermore, we detected no significant difference in response accuracy among the sessions over 4 days in mCherry mice (Figure 3D, day 2, 3, 4 vs. day 1, *p* = 0.37, 0.37, and 0.99, respectively). These results indicate that CNO application reversibly impaired attention by activation of hM4Di selectively expressed in the STN neurons, while CNO itself had no observable effect on response accuracy.

Although omission can also reflect inattentiveness (Robbins, 2002; Mar et al., 2013; Koike et al., 2015), no significant effect of CNO application was detected in both hM4Di and mCherry mice (Treatment × SD interaction, hM4Di, *F*_(3,21)_ = 1.41, *p* = 0.27; mCherry, *F*_(3,21)_ = 1.33, *p* = 0.29), and the percentage of omission remained similar between hM4Di and mCherry mice (Figures 3E,F). Because response accuracy is interpreted as a measure of sustained and spatially divided attention while omissions reflect global attentional processes in addition to motivation (Mar et al., 2013), our reversible chemogenetic manipulation of STN activity might have elucidated that the STN may directly be involved in specific subprocesses for attentional control.

### Decreased Accuracy Is Not Attributable to Dysfunction of Motor or Motivational Control

**Figure 4:**
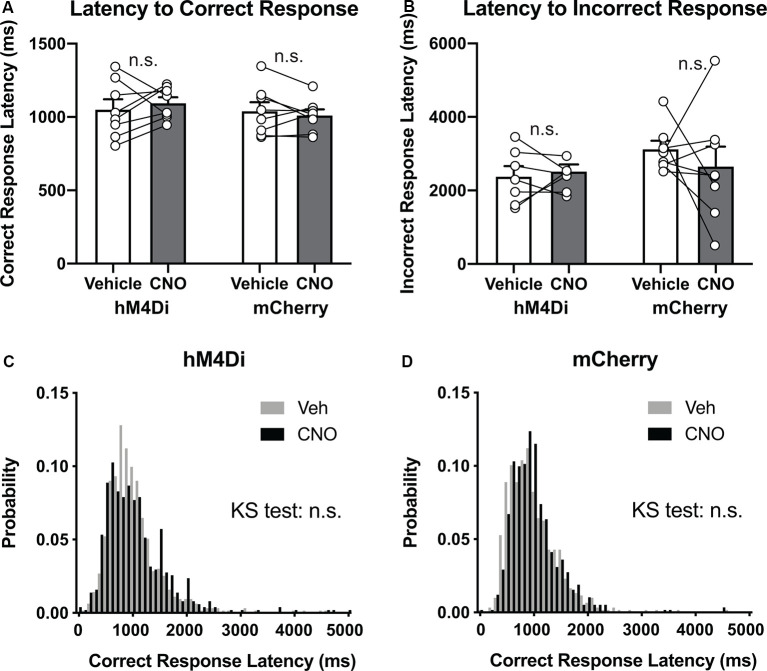
Decreased accuracy is not attributable to the dysfunction of motor control. **(A,B)** CNO treatment did not affect latency to correct response or latency to incorrect response in hM4Di mice and mCherry mice (*n* = 8 mice per group). **(C,D)** Histograms show the probability distribution of latencies to correct responses. n.s., not significant. Data are mean ± SEM.

Decreased response accuracy could also be attributed to impairment of motor or motivational control (Robbins, 2002). However, in comparison with vehicle treatment in both hM4Di and mCherry mice, CNO treatment in hM4Di mice did not affect latency to correct response (Figure 4A, Group × Treatment interaction, *F*_(1,14)_ = 0.99, *p* = 0.34) or latency to incorrect response (Figure 4B, *F*_(1,13)_ = 0.48, *p* = 0.50), which should be increased by motor impairment. No significant difference in the frequency distribution of the latency to correct response was observed between hM4Di and mCherry mice (Figures 4C,D, Vehicle vs. CNO in hM4Di, *p* = 0.51, Vehicle vs. CNO in mCherry, *p* = 0.93, KS-test). These data showed that CNO treatment in hM4Di mice had minimal effects on motor functions, at least in the context of 5-CSRTT.

Also, both hM4Di and mCherry mice completed the maximum number of trials in the presence or absence of CNO, CNO treatment did not affect latency to reward collection (Supplementary Figure S5, Group × Treatment interaction, *F*_(1,14)_ = 0.0013, *p* = 0.97), which should be increased under reduced motivation. Our data suggests that the effect of CNO on motivational control was minimal.

### Chemogenetic Reversible Suppression of the STN Neurons Increases Impulsivity but Not Compulsivity

**Figure 5:**
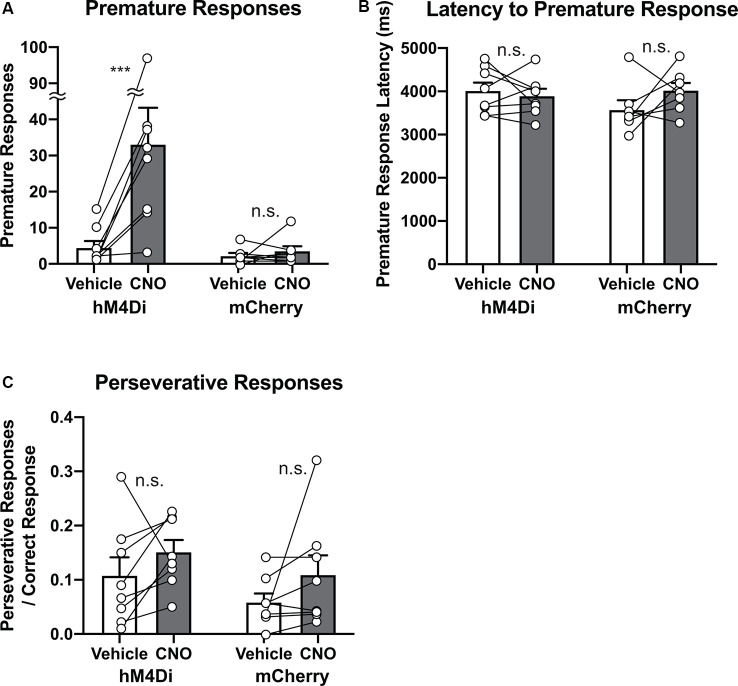
Chemogenetic reversible suppression of STN neurons increases impulsivity but not compulsivity. **(A)** CNO treatment significantly increased the number of premature responses in hM4Di mice but not mCherry mice (*n* = 8 mice per group). **(B)** CNO treatment did not affect latency to premature responses in hM4Di mice and mCherry mice. **(C)** CNO treatment did not affect perseverative responses after a correct response in hM4Di mice and mCherry mice. ***p < 0.001, n.s., not significant. Data are mean ± SEM.

Finally, to study whether chemogenetic suppression of STN activity affected response control, we analyzed premature and perseverative responses, which are regarded as measures of impulsivity and compulsivity, respectively (Robbins, 2002; Bari et al., 2008; Mar et al., 2013). As predicted from previous studies (Baunez and Robbins, 1997; Baunez et al., 2007; Frank et al., 2007), chemogenetic suppression of the STN activity significantly increased the number of premature responses (Figure 5A, Group × Treatment interaction, *F*_(1,14)_ = 9.65, *p* = 0.0077; Vehicle vs. CNO in hM4Di, *p* = 0.0008, Vehicle vs. CNO in mCherry, *p* > 0.99). In contrast, chemogenetic manipulation did not affect latency to premature responses (Figure 5B, Group × Treatment interaction, *F*_(1,13)_ = 2.31, *p* = 0.15). In the course of basic training, both the frequency and timing of premature responses were adaptively regulated (Supplementary Figure S3). CNO application selectively increased the number of premature responses but did not affect the latency to premature responses, suggesting that the occurrence tendency and onset timing of an impulsive response may discretely be controlled, and the STN may function as a part of the former system. As for compulsivity, we could not detect a significant effect of CNO treatment on perseverative responses in both hM4Di and mCherry mice (Figure 5C, Group × Treatment interaction, *F*_(1,14)_ = 0.026, *p* = 0.88). These results suggest that suppression of the STN activity increases impulsivity but not compulsivity, and the STN may be involved in suppressing an inappropriate response in a specific timing (e.g., delay period), but not in a general response control.

## Discussion

Because the STN is an obliquely oriented lens-shaped nucleus surrounded by several brain structures participating in cognitive processes (Mitrofanis, 2005; Zhang and van den Pol, 2017), the establishment of selective manipulation of the STN is critical for further dissection of its cognitive roles. To overcome difficulties in precise manipulation of defined neuronal populations, a combinatorial gene expression system using viral vectors and genetically-modified mouse strains presents a powerful solution. Pitx2 mRNA localizes within the STN and is distributed throughout the entire STN in adult mice (Martin et al., 2004; Schweizer et al., 2014, 2016). Furthermore, Pitx2-expressing glutamatergic neurons represent the vast majority of the STN subpopulation (Schweizer et al., 2016). AAV-DIO vector was stereotaxically injected into the Pitx2-Cre mouse brain, and DREADD expression was selectively introduced in the STN neurons (Figure 1), meaning that behavioral deficits induced by CNO administration are most likely not due to off-target effects. Furthermore, we conducted the electrophysiological analysis at a single-cell level and demonstrated that CNO treatment robustly decreases intrinsic excitability of the STN neurons by activation of hM4Di and thus suppresses the STN activity (Figure 2).

According to previous studies, attention is not a unitary process and includes several different types of subprocesses (Posner and Petersen, 1990). To assess how the STN activity is directly involved in attentional subprocesses, we conducted a 5-CSRTT. Chemogenetic silencing of the STN activity immediately and reversibly decreased accuracy, especially with shortened SD, but did not affect omission trials (Figure 3), suggesting that the STN is directly involved in information processing associated with sustained and divided attention required under highly demanding conditions, rather than global attention, which was reflected in the omission trials.

Recent behavioral studies using a 5-CSRTT have implicated the PFC and cholinergic neurons in the basal forebrain (BF) in attentional control (McGaughy et al., 2002; Chudasama et al., 2003; Ljubojevic et al., 2014; Koike et al., 2015; Kim et al., 2016). Within the PFC, the dorsal anterior cingulate cortex (dACC) is regarded as an essential area for attentional processing. Chemogenetic inactivation of dACC neurons affects both accuracy and omission in the 5-CSRTT (Koike et al., 2015). On the other hand, selective ablation of cholinergic cells in the BF decreases accuracy without affecting omission in the 5-CSRTT (McGaughy et al., 2002). Because one of the core functions of STN neurons is to relay cortical activity to subcortical brain structures (Alexander and Crutcher, 1990; Parent and Hazrati, 1995; Kolomiets et al., 2001; Nambu et al., 2002; Haynes and Haber, 2013; Kita et al., 2014), a subpopulation of STN neurons may receive signals associated with attentional processing from the dACC and transmit them to the BF. The PFC including the dACC densely projects to the STN (Kolomiets et al., 2001; Haynes and Haber, 2013; Kita et al., 2014). A recent transsynaptic retrograde tracing study using a replication-deficient rabies virus vector revealed that STN neurons directly project to cholinergic neurons in the BF (Gielow and Zaborszky, 2017). Taken together, our results and previously published findings suggest that information flowing from the dACC *via* the STN to the cholinergic system in the BF may play an important role in attention control reflected in the accuracy score in the 5-CSRTT.

Impulsivity and compulsivity are common properties of response control observed across species. We demonstrated that chemogenetic suppression of the STN activity reversibly increases premature response, which is regarded as an index for impulsivity in 5-CSRTT, but it does not affect compulsive responses (Figure 5), suggesting that the STN neurons actively control impulsivity, rather than compulsivity. As shown in Supplementary Figure S3, the number of premature responses and latencies to premature response change throughout basic training, suggesting that both the occurrence tendency and onset timing of an impulsive response are adaptively regulated. However, we found that inhibition of the STN activity selectively affected the number of premature responses but not the latencies to the premature responses. Our findings suggest that the occurrence tendency and onset timing of impulsive responses are separately controlled by different brain circuits, and the STN functions as a part of the former system.

Clinically, attentional impairments and enhanced impulsivity are observed as cognitive symptoms in some psychiatric disorders such as attention-deficit/hyperactivity disorder, schizophrenia, and addiction (American Psychiatric Association, 2013), and the dopaminergic system has been demonstrated to be deeply involved in the etiology of these disorders (Moeller et al., 2001). Because the STN is one of the major excitatory glutamatergic inputs to dopaminergic neurons in the SNc (Kanazawa et al., 1976; Iribe et al., 1999), it is also possible that the impairments in attention and response control immediately induced by chemogenetic suppression of the STN activity are mediated by dopaminergic modulation. Chemogenetic activation of the dopaminergic neurons or selective pharmacological enhancement of dopaminergic transmission induces attentional impairments and increased impulsivity in 5-CSRTT, in addition to motor dysfunction (Gaalen et al., 2006; Boekhoudt et al., 2017). On the other hand, it has been reported that electrical stimulation of the STN not only directly evokes monosynaptic excitatory responses but also induces polysynaptic inhibitory responses in the SNc dopaminergic neurons (Iribe et al., 1999). Furthermore, continuous manipulation of the STN activity critically alters the efficiency of excitatory synaptic transmission from the STN neurons to the SNc dopaminergic neurons (Ledonne et al., 2012). Taking these findings into consideration, chemogenetic suppression of the STN activity may dominantly decrease inhibitory synaptic events rather than excitatory events in a subpopulation of the dopaminergic neurons, and as a result, spatiotemporal changes in the dopaminergic modulation may affect cognitive processing in behavioral control. However, little is known about the dynamic relationship between STN activity and dopaminergic transmission. Future studies should elucidate how the STN neurons dynamically regulate the dopaminergic system by simultaneous measurement of neural activity in the STN and SNc during the cognitive tasks.

Because the STN receives inputs from motor cortical areas and DBS targeting the STN has been accepted as a therapy to treat motor symptoms of PD patients (Limousin et al., 1995; Benabid et al., 2009), the STN is thought to be profoundly involved in dynamic motor control. However, reversible and selective chemogenetic suppression of the STN activity did not induce apparent motor impairment, at least judging from the behavioral performance in 5-CSRTT (Figure 4). Therefore, further studies using challenging motor tasks are necessary to assess the functional roles of the STN in dynamic motor controls. In contrast, previous permanent lesion studies in rodents have demonstrated that the STN is involved in motor and non-motor cognitive functions (Baunez and Robbins, 1997, 1999; Baunez et al., 2007). Although the underlying mechanism is unknown, chronic blockade of the STN activity alters GABAergic synaptic transmission in the GP (Delfs et al., 1995), dopaminergic clearance in the Str (Schweizer et al., 2014), and trkB-dependent signaling in the SNc dopaminergic neurons (Luke Fischer et al., 2017), suggesting that the STN regulates circuit operation and plasticity in other basal ganglia components including GP, Str, and SNc. Because these basal ganglia components are postulated to be profoundly involved in both motor and non-motor cognitive functions, the STN may not only directly control information processing of these functions through their activity but also indirectly by modifying circuit property of other brain areas.

In this study, we demonstrated that chemogenetic inhibition of the STN activity immediately and reversibly impairs attention, which is especially required under highly demanding conditions and increases impulsivity but not compulsivity. Our findings, taken together with the results of previous lesion studies, suggest that the STN activity, directly and indirectly, participates in cognitive processing for controlling behavior, and dynamically regulates specific types of subprocesses in cognitive control directly through fast synaptic transmission. Selective and reversible manipulation of the STN neurons and their related brain areas using chemogenetic technology will be useful for understanding the biological basis of attention and response control, which are essential for cognitive processing in behavior, and also provide a powerful strategy to dissect the direct and indirect pathophysiological mechanisms of cognitive dysfunction in psychiatric and neurological disorders.

## Data Availability Statement

The raw data supporting the conclusions of this article will be made available by the authors, without undue reservation, to any qualified researcher.

## Ethics Statement

The animal study was reviewed and approved by the Animal Care and Use Committee of Kyoto University.

## Author Contributions

TN performed all the experiments and data analysis. KH and SY contributed to electrophysiology and data analysis. TH supported behavioral experiments. DW, TN, KH, and SY wrote the manuscript.

## Conflict of Interest

The authors declare that the research was conducted in the absence of any commercial or financial relationships that could be construed as a potential conflict of interest.
