# Supplementary material for: Chemogenetic Suppression of the Subthalamic Nucleus Induces Attentional Deficits and Impulsive Action in a Five-Choice Serial Reaction Time Task in Mice
Source: Front Syst Neurosci. 2020 Jun 30;14:38. doi: 10.3389/fnsys.2020.00038 (PMC7344274; doi:10.3389/fnsys.2020.00038)
Supplement: Supplementary file 1 [file Presentation_1.pdf]

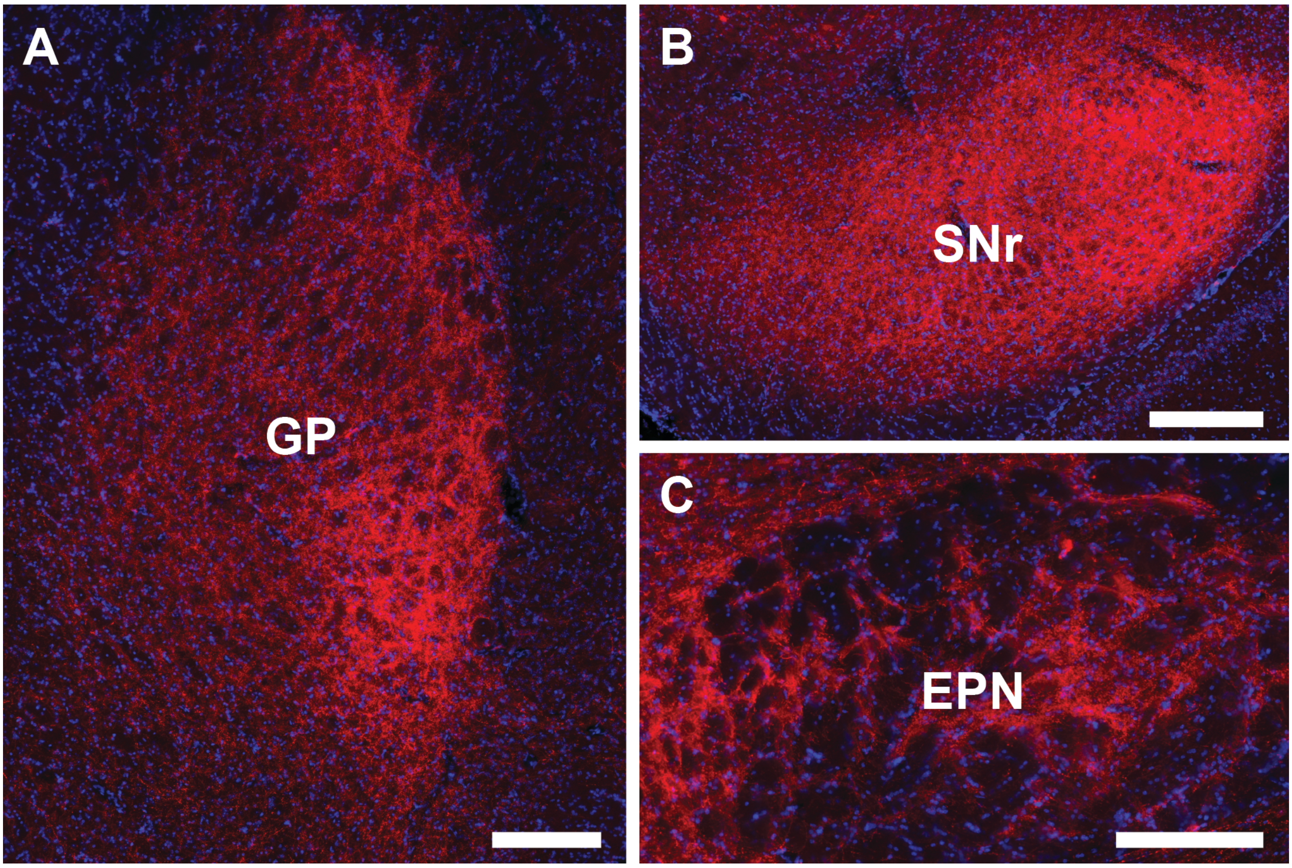

FIGURE S1 I Fluorescent reporter mCherry-positive neuropils were detected in the STN target structures after AAV injection into the Pitx2-Cre mouse brain. Images through STN terminal fields in **(A)** GP, **(B)** SNr, and **(C)** EPN. Scale bar, 200 µm. GP, globus pallidus; SNr, substantia nigra pars reticulata; EPN, entopeduncular nucleus.

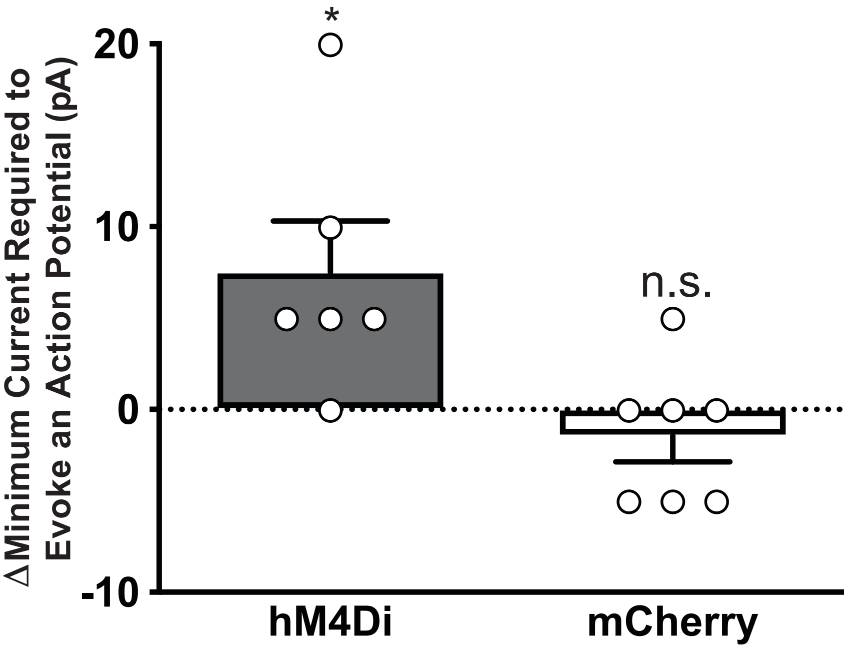

FIGURE S2 I CNO-induced activation of hM4Di suppresses the activity of STN neurons. CNO application increased the minimum current required to evoke an action potential of hM4Di-expressing cells but did not influence that of mCherry-expressing neurons. Group × Treatment interaction, $F_{(1,11)} = 8.77$, $^{*}p = 0.013$; before vs. after CNO in hM4Di, $^{*}p = 0.012$, before vs. after CNO in mCherry, $p > 0.99$, $^{*}p < 0.05$; n.s., not significant. Data are mean ± SEM.

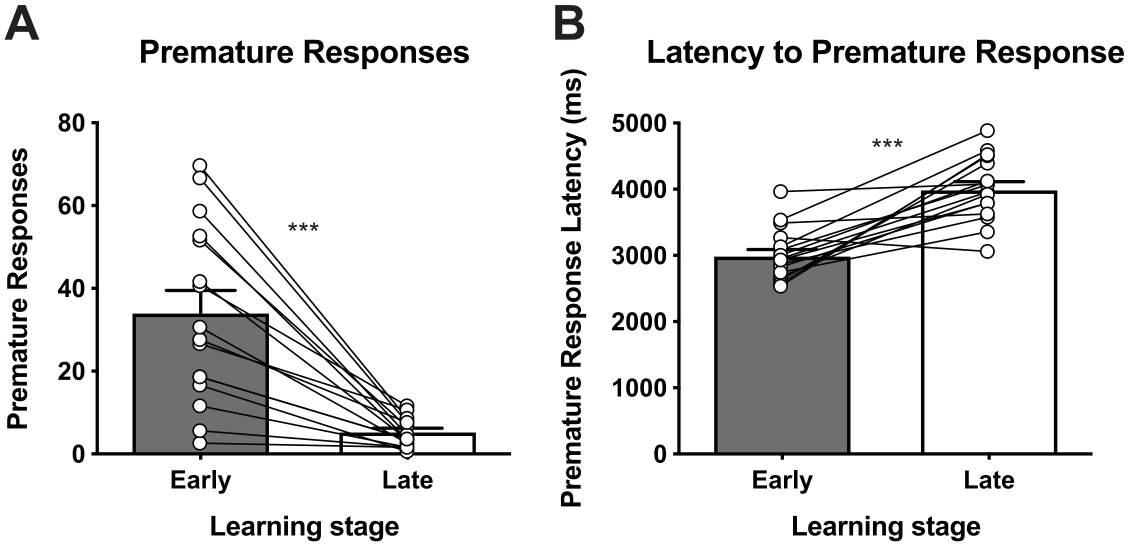

FIGURE S3 I The occurrence tendency and timing of impulsive responses were gradually controlled over training. **(A)** The number of premature responses significantly decreased over training. Early (the first session of the basic training) vs. Late (the last session of the basic training), $t_{(15)} = 5.82$, ***$p < 0.001$, two-tailed paired *t*-test. **(B)** Latency to premature response significantly increased over training. Early vs. Late, $t_{(15)} = 6.29$, ***$p < 0.0001$, Two-tailed paired *t*-test. Data are mean ± SEM.

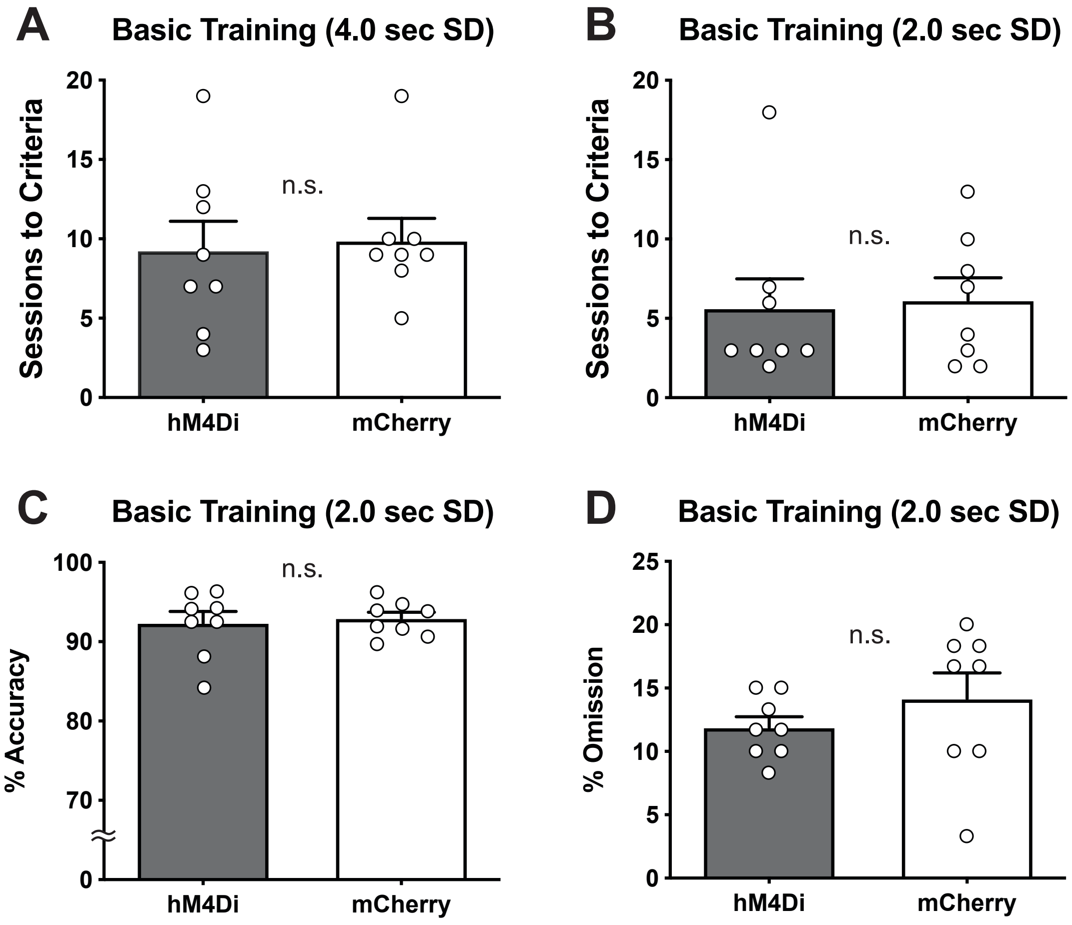

FIGURE S4 I The respective performances in basic training were similar between hM4Di and mCherry mice. **(A,B)** Sessions to criteria were not significantly different between two groups at 4.0 s or 2.0 s SD. hM4Di vs. mCherry at 4.0 s SD, $t_{(14)} = 0.27$, $p = 0.79$, Two-tailed unpaired *t*-test; hM4Di vs. mCherry at 2.0 s SD, $t_{(14)} = 0.21$, $p = 0.84$, Two-tailed unpaired *t*-test. **(C,D)** The baseline performances in hM4Di and mCherry mice were similar. Response accuracy in hM4Di mice vs. mCherry mice, $t_{(14)} = 0.33$, $p = 0.75$, Two-tailed unpaired *t*-test; Omission in hM4Di mice vs. mCherry mice, $t_{(14)} = 1.03$, $p = 0.32$, Two-tailed unpaired *t*-test. n.s., not significant, SD, stimulus duration. Data are mean ± SEM.

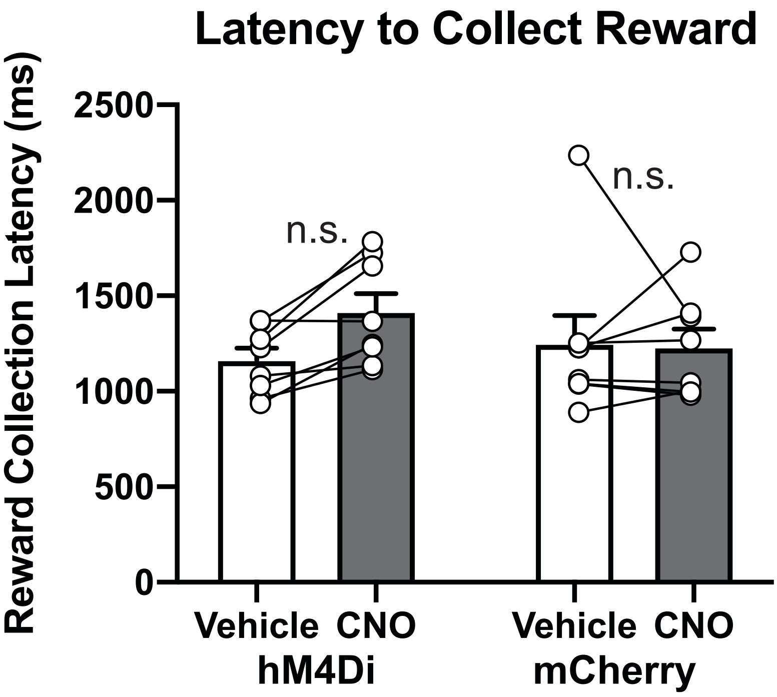

FIGURE S5 I Decreased accuracy was not attributable to the dysfunction of motivational control. CNO treatment did not affect latency to collect a reward in hM4Di mice and mCherry mice (*n* = 8 mice per group), n.s., not significant. Data are mean ± SEM.
